# Obsessive-compulsive disorder after traumatic brain injury: case report.

**DOI:** 10.1192/j.eurpsy.2023.1966

**Published:** 2023-07-19

**Authors:** R. M. Sousa, N. Cunha, P. Morgado

**Affiliations:** 1 Departamento de Psiquiatria e Saúde Mental, Centro Hospitalar Tondela-Viseu, Viseu; 2 Research Institute for Life and Health Sciences (ICVS), School of Medicine, University of Minho, Braga, Portugal

## Abstract

**Introduction:**

Although not the most prevalent clinical presentation, obsessive compulsive (OC) symptoms have been reported after TBI. Post-TBI OC disorder (OCD) cases are rare, so that OC symptoms in this setting are frequently described as OC personality disorders (OCPD).

Generally, the clinical features of post-TBI OCD are thought to be similar to those observed in idiopathic OCD, assuming the probable involvement of structures such as the orbitofrontal cortex, basal ganglia, limbic and thalamic systems in its pathophysiology, although no anatomical location clearly associated with post-TBI OCD being recognized.

**Objectives:**

Brief systematic review of OCD post-TBI and case report.

**Methods:**

Bibliographic research using Pubmed. Clinical interviews and file consultation, with patient informed consent.

**Results:**

We present a case of a 63-year-old patient referred to the Psychiatry Consultation due to obsessive thoughts of dirt and contamination, accompanied by compulsive cleaning and sanitizing behaviors with at least 3 years of evolution with a history of TBI and right frontopolar hemorrhage 5 years ago. These behaviors significantly impaired his functionality (cleaning objects on average 300 to 700 times a day, spending hours in the shower). The patient had insight for the excessive behaviors and its daily impairment.

**Conclusions:**

Psychopathology in the post-TBI context is not infrequent, however reported cases of post-TBI OCD are described as rare in the current literature. The short description of this phenomenon implies the need for more studies focused on the study of the phenomenology of post-TBI OCD. For example, while OCD and obsessive-compulsive symptoms tend to be recognizable psychiatric phenomena, neurobehavioral sequelae in a post-TBI context can present multiple manifestations and resemble OC phenomena, without actually constituting OCD.

**Disclosure of Interest:**

None Declared

